# Supplementary material for: Prevalence and consumption patterns of energy drinks among Iraqi adolescents: A cross-sectional study
Source: PLoS One. 2026 Mar 10;21(3):e0344654. doi: 10.1371/journal.pone.0344654 (PMC12974814; doi:10.1371/journal.pone.0344654)
Supplement: S2 File — Document describing the scoring method used to calculate socio-economic status of the participants. (DOCX) [file pone.0344654.s002.docx]

**Socio-economic Status**

**A scoring system was used to classify the adolescents according to their socio-economic status (SES). The total score (10) was divided into three categories: 0-3 (low), 4-6 (medium), and 7-10 (high).**

| **Variables** | **Categories** | **Score** | **Total score** |
| --- | --- | --- | --- |
| **Education level of the head of the family** | Illiterate | 0 | 4 |
|  | Can read and write, Primary | 1 |  |
|  | Intermediate school | 2 |  |
|  | High school or vocational | 3 |  |
|  | Bachelor's degree (college) and above | 4 |  |
| **Family income** | Not sufficient | 0 | 2 |
|  | Sufficient for daily needs | 1 |  |
|  | Exceeds for daily needs | 2 |  |
| **Occupation of head of the family** | Un-skilled workers | 0 | 2 |
|  | Lower professional | 1 |  |
|  | High professional | 2 |  |
| **Does your family have a private car?** | Yes | 1 | 1 |
|  | No | 0 |  |
| **Does your family own a house?** | Yes | 1 | 1 |
|  | No | 0 |  |
| **Total** |  | | 10 |
