# Supplementary material for: Prevalence and consumption patterns of energy drinks among Iraqi adolescents: A cross-sectional study
Source: PLoS One. 2026 Mar 10;21(3):e0344654. doi: 10.1371/journal.pone.0344654 (PMC12974814; doi:10.1371/journal.pone.0344654)

PLOS One

Prevalence and consumption patterns of energy drinks among Iraqi adolescents: A Cross-sectional study

--Manuscript Draft--

**Manuscript Number:**	PONE-D-26-00648
**Article Type:**	Research Article
**Full Title:**	Prevalence and consumption patterns of energy drinks among Iraqi adolescents: A Cross-sectional study
**Short Title:**	Prevalence and consumption patterns of energy drinks among Iraqi adolescents
**Corresponding Author:**	SORAN ABDULLAH Al-Keji, Ph.D<br>Hawler Medical University<br>Erbil, IRAQ
**Keywords:**	Energy drinks, Prevalence, Adolescence, Young adults, High school students
**Abstract:**	Background<br><br>Most energy drinks contain significant levels of caffeine, sugar, and other ingredients, which are consumed increasingly among adolescents. Consuming energy drinks is associated with various adverse health effects.<br><br>Objectives<br><br>To investigate the prevalence and pattern of energy drink consumption among high school students in Erbil City, The Kurdistan Region, Iraq.<br><br>Methods<br><br>A cross-sectional study was conducted from October 15th, 2024 to March 15th, 2025. A multistage cluster sampling technique was used to recruit 800 students from 20 selected High schools in Erbil, Iraq. SPSS version 27 was used for data entry and analysis, a p-value ≤0.05 is considered statistically significant.<br><br>Results<br><br>Overall, 82.1% of participants had reported at least a one-time-consumption of energy drinks in their lives, and 57.6% had consumed them in the past 30 days. The prevalence of energy drink consumption was significantly higher among males (58.3%, p=0.001). The study found a statistically significant association between education level of the head of the family, living arrangement, socioeconomic status and prevalence of energy drinks (p=0.001, p=0.023, p=0.006, respectively).<br><br>Conclusion<br><br>The prevalence of energy drinks consumption among high school aged adolescents in Iraq was high, and influenced by sociodemographic characteristics. Male students observed a statistically significant association with energy drink prevalence. The study underscores the need of the education intervention to enhance awareness among students on health risks associated with energy drink consumption.
**Order of Authors:**	SORAN ABDULLAH Al-Keji, Ph.D
	Samir Mahmood Othman
**Additional Information:**	
**Question**	**Response**
**Financial Disclosure**<br><br>Enter a financial disclosure statement that	The author(s) received no specific funding for this work.

describes the sources of funding for the work included in this submission. Review the submission guidelines for detailed requirements. View published research articles from *PLOS ONE* for specific examples.<br><br>This statement is required for submission and **will appear in the published article** if the submission is accepted. Please make sure it is accurate.<br><br>**Funded studies**<br>Enter a statement with the following details:<br>• Initials of the authors who received each award<br>• Grant numbers awarded to each author<br>• The full name of each funder<br>• URL of each funder website<br>• Did the sponsors or funders play any role in the study design, data collection and analysis, decision to publish, or preparation of the manuscript?<br><br>Did you receive funding for this work?	
**Competing Interests**<br><br>Use the instructions below to enter a competing interest statement for this submission. On behalf of all authors, disclose any competing interests that could be perceived to bias this work—acknowledging all financial support and any other relevant financial or non-financial competing interests.<br><br>This statement is required for submission and **will appear in the published article** if the submission is accepted. Please make sure it is accurate and that any funding sources listed in your Funding Information later in the submission form are also declared in your Financial Disclosure statement.<br><br>View published research articles from *PLOS ONE* for specific examples.	The authors have declared that no competing interests exist.

**NO authors have competing interests**<br><br>Enter: *The authors have declared that no competing interests exist.*<br><br>**Authors with competing interests**<br><br>Enter competing interest details beginning with this statement:<br><br>*I have read the journal's policy and the authors of this manuscript have the following competing interests: [insert competing interests here]*<br><br>* typeset	
**Ethics Statement**<br><br>Enter an ethics statement for this submission. This statement is required if the study involved:<br><br>• Human participants<br>• Human specimens or tissue<br>• Vertebrate animals or cephalopods<br>• Vertebrate embryos or tissues<br>• Field research<br><br>Write "N/A" if the submission does not require an ethics statement.<br><br>General guidance is provided below. Consult the submission guidelines for detailed instructions. **Make sure that all information entered here is included in the Methods section of the manuscript.**	The ethical approval was obtained from the College of Medicine Ethics Committee at Hawler Medical University, Kurdistan Region, Iraq (Code 9, September 2024). Every participant gave their verbal informed consent. The study did not involve any medical experimentation, and participation was completely voluntary and anonymous. Respondents were also notified that the questionnaire did not include any sensitive topics, that their replies would be kept anonymous, and that the obtained data would be used solely for research reasons. The Directorate of Education in Erbil receives permission to conduct the study. Parents' written consent was not sought because it was assumed that the students completed the questionnaire consciously.

Powered by Editorial Manager® and ProduXion Manager® from Aries Systems Corporation

**Format for specific study types**<br><br>**Human Subject Research (involving human participants and/or tissue)**<br>• Give the name of the institutional review board or ethics committee that approved the study<br>• Include the approval number and/or a statement indicating approval of this research<br>• Indicate the form of consent obtained (written/oral) or the reason that consent was not obtained (e.g. the data were analyzed anonymously)<br><br>**Animal Research (involving vertebrate animals, embryos or tissues)**<br>• Provide the name of the Institutional Animal Care and Use Committee (IACUC) or other relevant ethics board that reviewed the study protocol, and indicate whether they approved this research or granted a formal waiver of ethical approval<br>• Include an approval number if one was obtained<br>• If the study involved *non-human primates*, add *additional details* about animal welfare and steps taken to ameliorate suffering<br>• If anesthesia, euthanasia, or any kind of animal sacrifice is part of the study, include briefly which substances and/or methods were applied<br><br>**Field Research**<br><br>Include the following details if this study involves the collection of plant, animal, or other materials from a natural setting:<br>• Field permit number<br>• Name of the institution or relevant body that granted permission	
**Data Availability**<br><br>Authors are required to make all data underlying the findings described fully available, without restriction, and from the time of publication. PLOS allows rare exceptions to address legal and ethical concerns. See the PLOS Data Policy and FAQ for detailed information.	Yes - all data are fully available without restriction

A Data Availability Statement describing where the data can be found is required at submission. Your answers to this question constitute the Data Availability Statement and **will be published in the article**, if accepted.<br><br>**Important:** Stating 'data available on request from the author' is not sufficient. If your data are only available upon request, select 'No' for the first question and explain your exceptional situation in the text box.<br><br>Do the authors confirm that all data underlying the findings described in their manuscript are fully available without restriction?	
**Describe where the data may be found in full sentences. If you are copying our sample text, replace any instances of XXX with the appropriate details.**<br><br>• If the data are **held or will be held in a public repository**, include URLs, accession numbers or DOIs. If this information will only be available after acceptance, indicate this by ticking the box below. For example: *All XXX files are available from the XXX database (accession number(s) XXX, XXX.).*<br>• If the data are all contained **within the manuscript and/or Supporting Information files**, enter the following: *All relevant data are within the manuscript and its Supporting Information files.*<br>• If neither of these applies but you are able to provide **details of access elsewhere**, with or without limitations, please do so. For example:<br><br>*Data cannot be shared publicly because of [XXX]. Data are available from the XXX Institutional Data Access / Ethics Committee (contact via XXX) for researchers who meet the criteria for access to confidential data.*<br><br>*The data underlying the results presented in the study are available from (include the name of the third party*	All relevant data are within the manuscript and its Supporting Information files.

and contact information or URL).<br>• This text is appropriate if the data are owned by a third party and authors do not have permission to share the data.<br><br>* typeset	
Additional data availability information:	

Manuscript
Click here to access/download;Manuscript;Main Manuscript - .docx

Prevalence and consumption patterns of energy drinks among Iraqi adolescents: A Cross-sectional study

Soran Abdullah Al-keji[1¶], Samir Mahmood Othman[1¶]

[1]Department: Community Medicine, College of Medicine, Hawler Medical University, Erbil, Iraq

[2]Department: Community Medicine, College of Medicine, Hawler Medical University, Erbil, Iraq

* Corresponding author

E-mail: soranalkeji@gmail.com (SA)

[¶] SA performed the main analysis and drafted the manuscript.

[&] SM Supervised the study and critically revised the manuscript. Both authors approved the final version.

Abstract

Background: Most energy drinks contain significant levels of caffeine, sugar, and other ingredients, which are consumed increasingly among adolescents. Consuming energy drinks is associated with various adverse health effects.

Objectives: To investigate the prevalence and pattern of energy drink consumption among high school students in Erbil City, The Kurdistan Region, Iraq.

Methods: A cross-sectional study was conducted from October 15th, 2024 to March 15th, 2025. A multistage cluster sampling technique was used to recruit 800 students from 20 selected High schools in Erbil, Iraq. SPSS version 27 was used for data entry and analysis, a p-value ≤0.05 is considered statistically significant.

Results: Overall, 82.1% of participants had reported at least a one-time-consumption of energy drinks in their lives, and 57.6% had consumed them in the past 30 days. The prevalence of energy drink consumption was significantly higher among males (58.3%, p=0.001). The study found a statistically significant association between education level of the head of the family, living arrangement, socioeconomic status and prevalence of energy drinks (p=0.001, p=0.023, p=0.006, respectively).

Conclusion: The prevalence of energy drinks consumption among high school aged adolescents in Iraq was high, and influenced by sociodemographic characteristics. Male students observed a statistically significant association with energy drink prevalence. The study underscores the need of the education intervention to enhance awareness among students on health risks associated with energy drink consumption.

Introduction

Energy drinks are available in more than 140 countries, contributing to multibillion-dollar industry globally, which was initially introduced in Austria 1987 [1]. Energy drinks are promoted to increase energy, reduce symptoms of fatigue, and improve mental alertness and concentration. They contain significant levels of caffeine and sugar along with extra compounds including B vitamins, amino acids, and herbal stimulants like guarana [2].

Insomnia, anxiety, headaches, nausea, spontaneous miscarriage, tachycardia, increased platelet aggregation, and reduced endothelial function are linked to high caffeine and excessive energy drink usage [3]. The acute health effects include irritability, nervousness, tremors, insomnia, nausea, anxiety, cognitive disorders, diuresis, tachycardia, arrhythmia, fever, gastrointestinal disorders, elevated respiratory rate, and reduction of myocardial blood flow. Moreover, long-term exposure to caffeine is associated with severe health effects including a range of dysfunctions of the liver, gastrointestinal system, muscular system, and renal system[3,4]. Energy drink use has significantly increased during the last few decades, especially in Western and Asian nations. Youth-oriented media is frequently used to promote energy drinks [5].

Concerns about the general health and well-being of consumers have been highlighted by the rise in energy drink usage among teenagers and young adults. The constituents of energy drinks are often unknown to teenagers and young adults. Because energy drinks include high amounts of caffeine and other substances that are not often present in food, they pose a real health risk to the general public, especially to young people [6].

Students are a segment of the population that is very susceptible to stress, which is mostly caused by their lifestyle, studies, and tests. Thus, they are more likely to consume energy drinks containing caffeine and other unusual ingredients. Even more concerning, the majority of them are unaware of these drinks' harmful adverse effects. The energizing, deceptive effects of energy drinks make people feel more alert than they actually are.[7]. The most commonly acknowledged reasons consuming energy drink include: studying for an exam, preparing a project, and staying alert. Previous studies have found a positive correlation between perceived stress and energy drink use; this association between

academic performance, perceived stress, and energy drink intake suggests that younger students may become more dependent on energy drinks [8].

Consumption of energy drinks has grown dramatically in recent years. Teenagers, athletes, and students are among the most frequent customers for various reasons. Unfortunately, just a few studies have been published to investigate the impact of these beverages on health and cognitive performance [9]. Energy drink usage is prevalent among adolescents and school students, and has become a topic of interest in recent decades, with varying rates across countries worldwide. The lowest prevalence was reported from Finland, 24.4% [10], and 31.3% in Hungary [11], and the highest prevalence were reported in Congo, 63% [12], and 66% in America [13]. In the Gulf Countries, which have a similar cultural and sociodemographic relation to Iraq, there was a prevalence of 45-60% among both middle and secondary school students [14]. In Iraq, only one study has been conducted in different Middle Euphrates Cities, among college students, fitness center users, and public place attendants, not from school. The prevalence of monthly energy drink consumption was 50.2% [15]. Bearing in mind the large number of high school students in Iraq and the several types of affordable and readily available options for them underscores the need for an estimation of the prevalence of consumption among high school students, and especially its relationship with the educational background of the parents and the sociodemographic factors affecting the consumption of energy drinks among adolescents in Iraq.

There is limited information available on energy drinks among high school students in Erbil, the capital of the Kurdistan Region of Iraq. This study aims to assess the prevalence and consumption patterns of energy drinks among high school students in Erbil City, Kurdistan Region of Iraq.

Material and methods

Study design, sample size, and sampling method

A school-based cross-sectional study was conducted in Erbil, Iraq, from October 15th, 2024, to March 15th, 2025. Before starting data collection, the consent form was obtained from the schools' administrations, and convenient dates were scheduled. Epi Info version

7 was used to estimate the sample size by using a confidence interval of 95%, 5% degree of precision, estimated frequency of 42.7 % from the other study done in the region[7], population size of 29013, and design effect 2; the estimated sample size was 732. A multistage cluster sampling technique was used for selecting schools and students. In the first stage, a sample of 800 high school students from 20 schools out of 72 high schools in Erbil city by using the probability proportional size sampling technique. In the second stage, a stratified random sampling method was used to select students in the selected schools. A modified self-administered questionnaire was used to collect data from students of both sexes to assess the prevalence and consumption patterns of energy drinks[16–18].

Data collection tool

The questionnaire included closed-ended and multiple-choice questions, organized into two sections. The first section includes the sociodemographic characteristics. The second section evaluates energy drink consumption patterns among participants. Questions related to energy drinks included: place of purchase, time of consumption, consumption patterns, and frequency of consumption. The questionnaire was designed to address the study's questions related to the aim and objectives, initially in English and translated into the local languages (Kurdish and Arabic).

Ethical considerations

Before starting the study, the ethical approval was obtained from the College of Medicine Ethics Committee at Hawler Medical University, Kurdistan Region, Iraq (Code 9, September 2024). Every participant gave their verbal informed consent. The study did not involve any medical experimentation, and participation was completely voluntary and anonymous. Respondents were also notified that the questionnaire did not include any sensitive topics, that their replies would be kept anonymous, and that the obtained data would be used solely for research reasons. The Directorate of Education in Erbil receives permission to conduct the study. Parents' written consent was not sought because it was assumed that the students completed the questionnaire consciously.

Statistical analysis

Data were entered into a computer using the Statistical Package for the Social Sciences (SPSS, version 27). Descriptive statistics were useded to calculate frequencies and percentages, whereas analytical statistics employed Pearson's Chi-square test to analyze the relationship between categorical variables such as gender, age, and living arrangement and energy drink use prevalence. A p-value < 0.05 indicates statistical significance.

Results

Socio-demographic characteristics

Table 1 shows the socio-demographic characteristics of the research respondents. In terms of the academic level of the participants among the 800 students who completed the questionnaire, the highest proportion belonged to 10th grade (379 students, 44.2%), the lowest was 12th grade (191 students, 21.7%), in comparison the rest were 11th grade (251 students, 28.5%). The study sample had a slightly higher proportion of female students than male students. Females comprised of 57.3% (n = 458) of the participants, while males accounted for 42.7% (n = 342). The age of the high school students in the selected sample ranged from 15 to 20 years, with a mean age of approximately 16.5 years and a standard deviation of 1.191, indicating that most participants' ages were clustered reasonably close to the mean. The majority of the participants were 16 years old, accounting for 29.3% (n=234) of the total study sample, and the least age group was 20 years old, with only 1.4% (n=11) of the sample.

Regarding participants’ Religion, the vast majority of the respondents were Muslim, accounting for 795 students (99.4%). The sample’s living arrangement showed that a substantial majority, 95% (n=760), reported living with both parents. A small proportion of participants lived with a single parent, 3.8% (n=30), while only 1.3% (n=10) lived with others.

The distribution of participants by Ethnicity indicates that the predominant proportion of respondents, 94.6% (n=755), were Kurdish. A small proportion of participants were Arab, 2.9% (n=23), and Turkish, 2.8% (n=22), respectively. The education level of the head of the family in the study sample showed that the most common level was completed primary school 44.1% (n=353), and fewer participants reported that their head of family had completed high school or was illiterate (12%).

Moreover, the families' monthly incomes showed that 53.9% (n=431) of the respondents stated that their family's monthly income was sufficient for daily needs, and only 17.5% (n=140) reported that it was not sufficient for their daily lives. These results suggested that most of the study sample lived in a family with at least a basic level of economic stability. The occupation of the head of family of the participants showed that 64.5% (n=516), of the study sample, stated that their household earners worked in lower professional roles, and 12.6% (n=101) came from households where the main occupation was unskilled work. This distribution of the data indicated that most of the study respondents came from middle-level occupational backgrounds.

The distribution of the study sample by socio-economic status demonstrated that the majority of the participants, 54.9% (n=439) were classified as having a medium socio-economic status. After that, 35.1% (n=281) fell within the high-level category, while only 10% (n=80) were categorized as the low level. This distribution indicates that a smaller proportion of the participants lived in low-income conditions, and most of the respondents were from middle- to upper income backgrounds.

Table 1: Socio-demographic characteristics of study respondents

**Socio-demographic characteristics**		**No.**	**%**
**Academic Level**	10	379	47.4
	11	241	30.1
	12	180	22.5

**Sex**	Male	342	42.8
	Female	458	57.3
**Age**	15	184	23.0
	16	234	29.3
	17	222	27.8
	18	115	14.4
	19	35	4.3
	20	10	1.4
**Religion**	Muslim	795	99.4
	Christian	3	.4
	Kakayi	2	.3
**Living Arrangement**	With both parents	760	95.0
	with a single parent	30	3.8
	Others	10	1.3
**Ethnicity**	Kurdish	755	94.4
	Arabic	23	2.9
	Turkish	22	2.8
**Education level of the head of the family**	Illiterate	44	5.5
	Can read and write, Primary	353	44.1
	Intermediate school	96	12.0
	High school or vocational	78	9.8
	Bachelor's degree (college) and above	229	28.6
**Family income**	Not sufficient	140	17.5
	Sufficient for daily needs	431	53.9
	Exceeds daily needs	229	28.6
	Unskilled workers	101	12.6

**Occupation of head of family**	Lower professional	516	64.5
	High professional	183	22.9
**Socio-economic status**	Low	80	10.0
	Medium	439	54.9
	High	281	35.1

Lifetime prevalence of Energy drinks consumption

(Fig 1) demonstrates the lifetime prevalence of energy drink consumption among the study sample. The majority of participants, 82.1% (n=657), reported consuming energy drinks at some point in their lives, in comparison, only 17.9% (n=143) stated that they had never consumed energy drinks. This result suggested a relatively high lifetime prevalence of energy drink consumption among high school students.

Fig 1 Lifetime prevalence of energy drink consumption

Prevalence of energy drink consumption

Figure 2 displays the previous month's prevalence of energy drink consumption among high school students in Erbil, Iraq. More than half of the participants,57.6% (n=461), reported consuming energy drinks in the previous month, while 42.4% (n=339) had not consumed any during the same period. This finding indicates a very high current consumption rate, suggesting that energy drink consumption is common among high school students.

Fig 2 Prevalence of energy drink consumption

Association of sociodemographic characteristics and prevalence of energy drink consumption

Table 2 shows the relationship between the previous month's energy drink use and the research sample's socio-demographic characteristics.

The analysis includes 800 respondents. Among the participants, 461 (57.6%) reported using energy drinks in the month prior to data collection, whereas 339 did not.

The analysis found that there are significant associations between several socio-demographic factors. Notably, gender was significantly associated with energy drink consumption ($p < 0.001$), with a higher proportion of male students (50.8%) reporting consumption compared to female students (49.2%). However, this difference is considered slight. Similarly, a statistically significant association ($p = 0.006$) with socio-economic status was observed in the analysis, with higher energy drink consumption reported among respondents from a medium SES background compared to both high (43.1%) and low (9.7%).

Additionally, the education level of the head of the family demonstrated a strong, statistically significant association with energy drink consumption ($p < 0.001$). Householders with a primary education level who can read and write reported a higher consumption rate of 50.8% than those with other education levels.

Moreover, Living Arrangement was significant associated with energy drink intake ($p = 0.023$). Participants who live with both parents are more likely to consume energy drinks than those living with one parent, 5.2%, or in other living arrangements, 0.9%.

In contrast, no significant statistical associations were found between energy drinks and some variables, such as participant education level ($p = 0.270$), Religion ($p = 0.929$), Ethnicity ($p = 0.119$), Family income ($p = 0.057$), and occupation of the head of the family ($p = 0.353$).

These findings indicate that specific socio-demographic characteristics, particularly gender, the education level of the head of the family, and socio-economic status, may

influence the likelihood of energy drink consumption among high school students in Erbil city of the Kurdistan region of Iraq.

Table 2: The relationship between the prevalence of energy drink intake and sociodemographic variables

		Have you consumed any energy drinks in the previous month?				P-value
		Yes		No		
		No.	%	No.	%	
**Academic level**	10	220	47.7	159	46.9	0.270
	11	146	31.7	95	28.0	
	12	95	20.6	85	25.1	
**Sex**	Male	234	50.8	108	31.9	**<0.001**
	Female	227	49.2	231	68.1	
**Religion**	Muslim	458	99.3	337	99.4	0.929
	Christian	2	.4	1	.3	
	Kakayi	1	.2	1	.3	
**Living Arrangement**	With both parents	433	93.9	327	96.5	**0.023**
	with a single parent	24	5.2	6	1.8	
	Others	4	.9	6	1.8	
**Ethnicity**	Kurdish	439	95.2	316	93.2	0.119
	Arabic	14	3.0	9	2.7	
	Turkish	8	1.7	14	4.1	
**Education level of the head of the family**	Illiterate	28	6.1	16	4.7	**<0.001**
	Can read and write, Primary	234	50.8	119	35.1	
	Intermediate school	47	10.2	49	14.5	
	High school or vocational	31	6.7	47	13.9	
	Bachelor's degree (college) and above	121	26.2	108	31.9	
**Family income**	Not sufficient	75	16.3	65	19.2	0.057
	Sufficient for daily needs	265	57.5	166	49.0	
	Exceeds for daily needs	121	26.2	108	31.9	
	Unskilled workers	60	13.0	41	12.1	0.353

**Occupation of head of family**	Lower professional	304	65.9	212	62.5	
	High professional	97	21.0	86	25.4	
**SES**	Low	47	10.2	33	9.7	**0.006**
	Medium	273	59.2	166	49.0	
	High	141	30.6	140	41.3	

Discussion

In this study, the participants' socio-demographic characteristics provided an important context for interpreting the outcomes of energy drink consumption among high school students in Erbil, Iraq. Females comprised higher percentage of the study sample in comparison to males. This figure consistent with a previous study, which argued that gender variation could be found in energy drink consumption [19]. However, these minor differences may be produced by the high number of female's schools in the target population of the study and the sampling method, which was probability proportional to size.

The demographic characteristic of the study sample shows relatively a homogenous sample in terms of Ethnicity, Religion, and family income stability, with slight variation in socioeconomic status. These features of the sample are important for the generalizability of research findings. In addition, it may influence the effectiveness of intervention strategies aimed at reducing energy drinks among adolescents and the consumption patterns of energy drinks.

The findings of the study underscore a high lifetime prevalence of energy drinks among adolescents in Iraq. This lifetime prevalence is higher than the estimated worldwide prevalence of 54.7% [20]. Additionally, it is higher than a similar study conducted in Germany, which stated a prevalence rate of 61.7% [21]. Given the high lifetime prevalence of energy drinks, the recommendation of an energy drink reduction education program for the high school population has strong potential to yield measurable public health benefits.

Moreover, the significant findings of the study were estimating the prevalence of energy drinks, which was 57.6%, indicating that a considerable proportion of the respondents consumed energy drinks in the prior month. It also represents a widespread and frequent energy drink consumption among adolescents in Erbil, Iraq. This current prevalence is significantly higher than the worldwide prevalence over the past 30 days, at 21.6% [20]. Additionally, it is also greater than findings from a study conducted in Saudi Arabia among university students, which reported that 27.2 of the university students consumed at least one beverage per month [22].

Regarding the relationship between the sample's sociodemographic variables and the prevalence of energy drink use in the previous month. The investigation indicated a statistically significant relationship between gender and energy drink intake.

Regarding the relationship between the sample's sociodemographic variables and the previous month's prevalence of energy drink usage. The analysis indicated a statistically significant relationship between gender and energy drink usage ($p < 0.001$). Male respondents reported greater rates of energy drink intake than females. This finding is consistent with prior research indicating gender differences in energy drink consumption, with males often demonstrating higher consumption, may be due to cultural, social, and behavioral factors, for instance, cultural norms that discourage such behaviors for females, and females who drink in public are often judged more negatively than men, being perceived as less respectable[19,23,24].

Additionally, the academic level of the head of the family of participants was associated with energy drink consumption in a statistically significant manner ($p < 0.002$). Participants from households with lower educational attainment report higher consumption than those from households headed by family members with higher educational attainment. This pattern of the study's results aligns with a study in the United Arab Emirates, reporting that adolescents from parents with a higher-educated background are generally associated with greater awareness of the health risks associated with energy drink consumption [25].

Moreover, a statistically significant correlation was found between individuals' living arrangements and the use of energy beverages ($p = 0.023$). Participants living with a single

parent reported lower energy drink consumption compared to those living with both parents. This may indicate cultural and social practices of shared meals and family gatherings among participants in Kurdish culture in Erbil city that involve energy drink consumption. This statement is similar to the study findings, which indicated that the living arrangement of the students significantly influenced the consumption of energy-dense and nutrient-poor foods. More specifically, students living with their parents reported higher consumption of energy drinks and nutrient-poor foods, including snacks, fast food, and sugary drinks. In contrast this pattern of energy drinks and food differs from studies' findings in the USA, Europe, and the UK, where students living with their parents reported consuming fewer energy drinks and nutrient-poor foods [26].

Additionally, students' socioeconomic status demonstrated a statistically significant association with the prevalence of energy drink consumption. In particular, students who live in lower socioeconomic conditions are more likely to consume energy drink than those live in higher socioeconomic status. This finding is in line with a study argued that individuals from low socioeconomic status were independently associated with high daily energy drink consumption[16]. A clarification of this relationship is that consumption of energy drinks is associated with markers of low health education and unhealthy behaviors, which are mostly related to low levels of socioeconomic status [27].

Conclusion

The study provides considerable evidence on the consumption patterns of energy drinks among youth and adolescents in Erbil, Iraq. It also assesses the influence of sociodemographic factors on energy drink consumption. More than half of the participants reported consuming energy drinks in the previous 30 days. Additionally, this study found that the prior-month prevalence of energy drinks was statistically significantly associated with males. Furthermore, it highlights the need to introduce appropriate and stricter regulations on the marketing and sale of energy drinks, particularly to adolescents and young adults to curb their widespread availability. The high prevalence of energy drinks among high school students underscores the crucial need for environmental control, policy

measures, and educational intervention to reduce energy drink consumption among adolescents in Iraq.

Limitations

The study provides valuable insights into the frequency and patterns of energy drink intake among Iraqi adolescents. The study's reliance on self-reported data may introduce recall and social desirability biases.

Conflict of Interest Statement

The research was conducted in the absence of any financial relationships or commercial interests that could be construed as a potential conflict of interest.

Acknowledgments

I acknowledge the education members of the community health department of the College of Medicine in Hawler Medical University and my colleagues for their support, and appreciate the staff of the Department of Statistics of the Directorate of Education of Erbil for their outstanding cooperation.

References

1. Ali F, Rehman H, Babayan Z, Stapleton D, Joshi DD. Energy drinks and their adverse health effects: A systematic review of the current evidence. Postgraduate Medicine. 2015 May 4;127(3):308–322.

2. Al-Shaar L, Vercammen K, Lu C, Richardson S, Tamez M, Mattei J. Health Effects and Public Health Concerns of Energy Drink Consumption in the United States: A Mini-Review. Front Public Health [Internet]. 2017 Aug 31 [cited 2025 Dec 18];5. Available from: https://www.frontiersin.org/journals/public-health/articles/10.3389/fpubh.2017.00225/full

3. Ehlers A, Marakis G, Lampen A, Hirsch-Ernst KI. Risk assessment of energy drinks with focus on cardiovascular parameters and energy drink consumption in Europe. Food and Chemical Toxicology. 2019 Aug 1;130:109–121.

4. Breda JJ, Whiting SH, Encarnação R, Norberg S, Jones R, Reinap M, et al. Energy Drink Consumption in Europe: A Review of the Risks, Adverse Health Effects, and Policy Options to Respond. Front Public Health [Internet]. 2014 Oct 14 [cited 2025 Dec 20];2. Available from: https://www.frontiersin.org/journals/public-health/articles/10.3389/fpubh.2014.00134/full

5. de Sanctis V, Soliman N, T Soliman A, Elsedfy H, Di Maio S, El Kholy M, et al. Caffeinated energy drink consumption among adolescents and potential health consequences associated with their use: a significant public health hazard. Acta Biomed. 2017;88(2):222–231.

6. Chandrasekaran V, Shide E, author(s) additional. The effects of energy drinks on the structure and function of epithelial cells and fibroblasts. West Coast Biological Science Undergraduate Research Conference. 2011 Jan 1;1–19.

7. Asaad YA. Energy drinks consumption in Erbil city: A population based study. Zanco Journal of Medical Sciences (Zanco J Med Sci). 2017 Aug 29;21(2):1680–1687.

8. Poulos, Pasch. Socio-demographic differences in energy drink consumption and reasons for consumption among US college students - Natalie S Poulos, Keryn E Pasch, 2016 [Internet]. Sage Journals. [cited 2025 Dec 19]. Available from: https://journals.sagepub.com/doi/abs/10.1177/0017896915578299

9. Chang YJ, Peng CY, Lan YC. Consumption of Energy Drinks among Undergraduate Students in Taiwan: Related Factors and Associations with Substance Use. International Journal of Environmental Research and Public Health. 2017 Sept;14(9):954.

10. Puupponen M, Tynjälä J, Tolvanen A, Välimaa R, Paakkari L. Energy Drink Consumption Among Finnish Adolescents: Prevalence, Associated Background Factors, Individual Resources, and Family Factors. Int J Public Health. 2021 May 7;66:620268.

11. Tóth Á, Soós R, Szovák E, M. Najbauer N, Tényi D, Csábí G, et al. Energy Drink Consumption, Depression, and Salutogenic Sense of Coherence Among Adolescents and Young Adults. International Journal of Environmental Research and Public Health. 2020 Jan;17(4):1290.

12. Carsi Kuhangana T, Muta Musambo T, Pyana Kitenge J, Kayembe-Kitenge T, Kazadi Ngoy A, Musa Obadia P, et al. Energy Drink Consumption among Adolescents Attending Schools in Lubumbashi, Democratic Republic of Congo. International Journal of Environmental Research and Public Health. 2021 Jan;18(14):7617.

13. Miller KE, Dermen KH, Lucke JF. Caffeinated energy drink use by U.S. adolescents aged 13–17: A national profile. Psychology of Addictive Behaviors. 2018;32(6):647–659.

14. Faris ME, Al Gharaibeh F, Islam MR, Abdelrahim D, Saif ER, Turki EA, et al. Caffeinated energy drink consumption among Emirati adolescents is associated with a cluster of poor physical and mental health, and unhealthy dietary and lifestyle behaviors: a cross-sectional study. Front Public Health [Internet]. 2023 Oct 16 [cited 2025 Dec 19];11. Available from: https://www.frontiersin.org/journals/public-health/articles/10.3389/fpubh.2023.1259109/full

15. Mekkey SM, Tizkam HH. The Prevalence Rate of Energy Drinks Consumption Among Young Adults in Iraqi Society. Medical Journal of Babylon. 2022 Sept;19(3):488.

16. Degirmenci N, Fossum IN, Strand TA, Vaktskjold A, Holten-Andersen MN. Consumption of energy drinks among adolescents in Norway: a cross-sectional study. BMC Public Health. 2018 Dec 19;18(1):1391.

17. Magnezi R, Bergman LC, Grinvald-Fogel H, Cohen HA. A survey of energy drink and alcohol mixed with energy drink consumption. Isr J Health Policy Res. 2015 Dec 1;4(1):55.

18. Musaiger AO, Zagzoog N. Knowledge, Attitudes and Practices toward Energy Drinks among Adolescents in Saudi Arabia. Glob J Health Sci. 2014 Mar;6(2):42–46.

19. Lebacq T, Desnouck V, Dujeu M, Holmberg E, Pedroni C, Castetbon K. Determinants of energy drink consumption in adolescents: identification of sex-specific patterns. Public Health. 2020 Aug 1;185:182–188.

20. Aonso-Diego, Krotter, García-Pérez. Prevalence of energy drink consumption world-wide: A systematic review and meta-analysis - Aonso-Diego - 2024 - Addiction - Wiley Online Library [Internet]. Wiley Online Library. 2024 [cited 2025 Dec 18]. Available from: https://onlinelibrary.wiley.com/doi/full/10.1111/add.16390

21. Galimov A, Hanewinkel R, Hansen J, Unger JB, Sussman S, Morgenstern M. Energy drink consumption among German adolescents: Prevalence, correlates, and predictors of initiation. Appetite. 2019 Aug 1;139:172–179.

22. Bawazeer NA, AlSobahi NA. Prevalence and Side Effects of Energy Drink Consumption among Medical Students at Umm Al-Qura University, Saudi Arabia. International Journal of Medical Students. 2013 Dec 31;1(3):104–108.

23. Alabbad M, AlMussalam M, AlMusalmi A, Alealiwi M, Alresasy A, Alyaseen H, et al. Determinants of energy drinks consumption among the students of a Saudi University. J Fam Community Med. 2019;26(1):36.

24. Ghozayel M, Ghaddar A, Farhat G, Nasreddine L, Kara J, Jomaa L. Energy drinks consumption and perceptions among University Students in Beirut, Lebanon: A mixed methods approach. PLOS ONE. 2020 Apr 30;15(4):e0232199.

25. Almulla AA, Faris MAIE. Energy Drinks Consumption Is Associated With Reduced Sleep Duration and Increased Energy-Dense Fast Foods Consumption Among School Students: A Cross-Sectional Study. Asia Pac J Public Health. 2020 July 1;32(5):266–273.

26. Whatnall, Patterson, Chiu, Oldmeadow, Hutchesson. Determinants of eating behaviours in Australian university students: A cross-sectional analysis - Whatnall - 2020 - Nutrition & Dietetics - Wiley Online Library [Internet]. Wiley Online Library. 2019 [cited 2025 Dec 20]. Available from: https://onlinelibrary.wiley.com/doi/abs/10.1111/1747-0080.12584

27. Svensson Å, Warne M, Gillander Gådin K. Longitudinal Associations Between Energy Drink Consumption, Health, and Norm-Breaking Behavior Among Swedish Adolescents. Front Public Health [Internet]. 2021 June 11 [cited 2025 Dec 19];9. Available from: https://www.frontiersin.org/journals/public-health/articles/10.3389/fpubh.2021.597613/full

Click here to access/download;Figure;Figure1.tiff

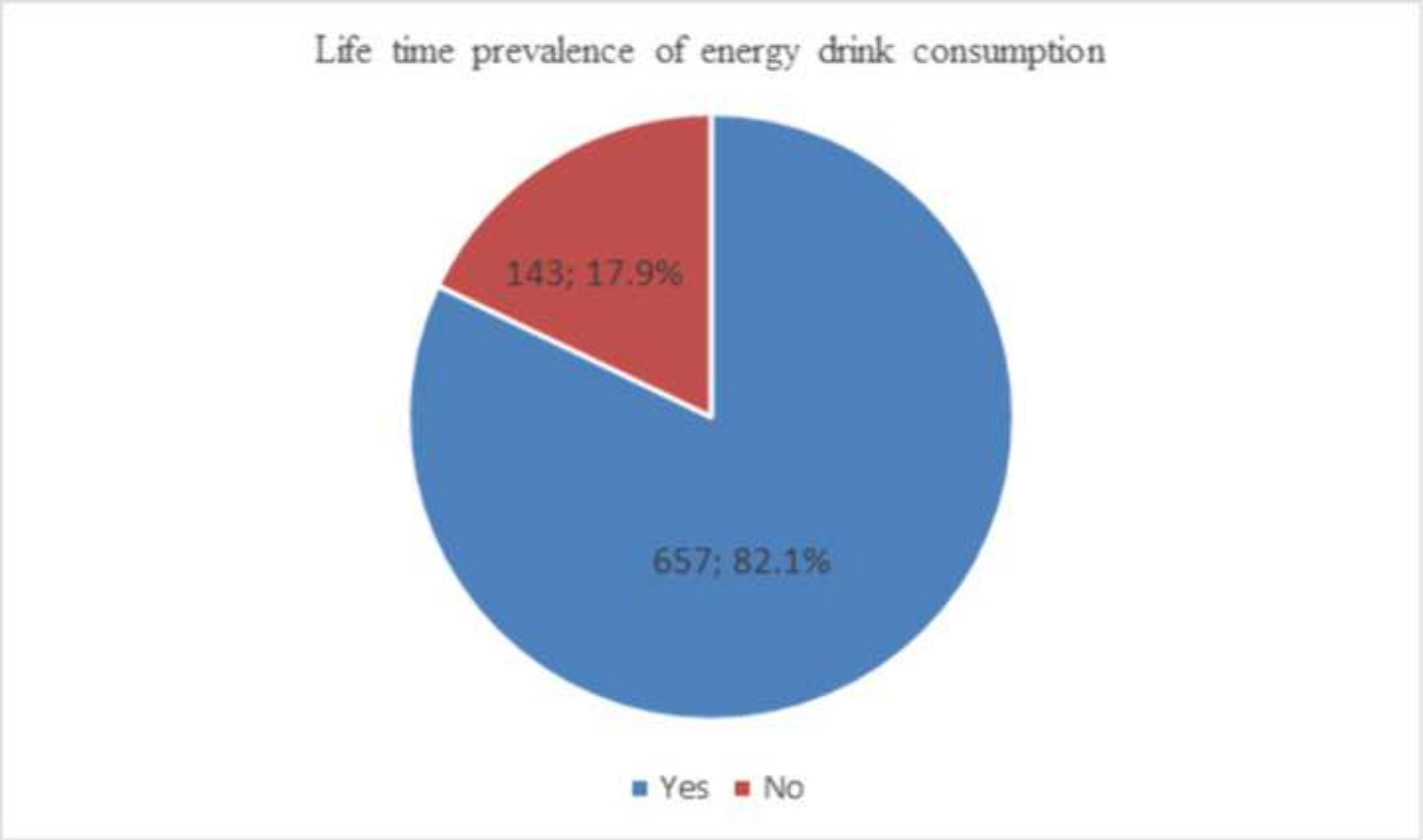

Figure 2

Click here to access/download;Figure;Figure2.tiff

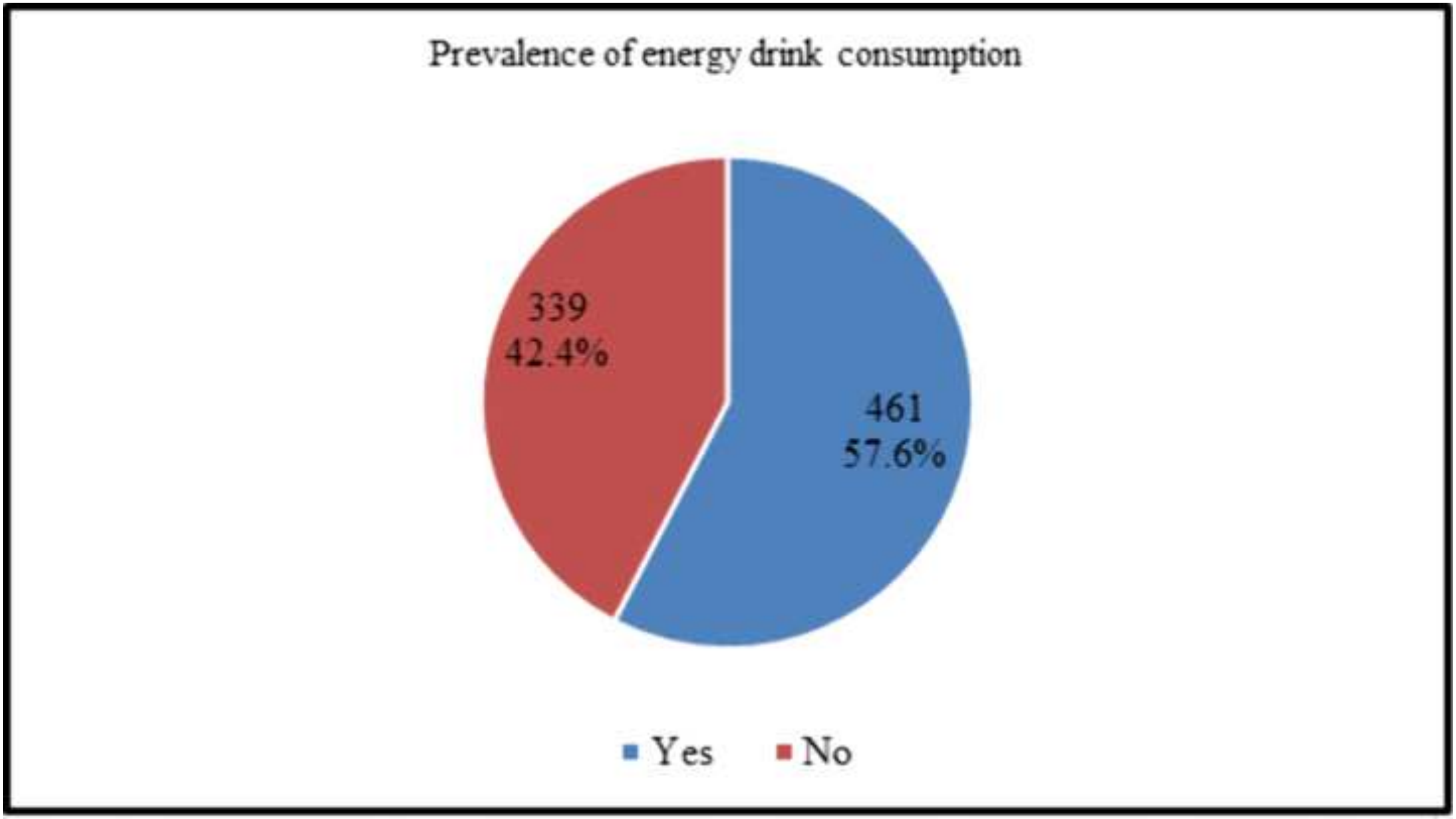

Supplement: Attachment — Submitted filename: F PONE-D-26-00648.pdf [file pone.0344654.s003.pdf]
